# Case report: A pediatric case of repeated false-positive urea breath test for *Helicobacter pylori* without decreased gastric acid secretion

**DOI:** 10.3389/fmed.2023.1267180

**Published:** 2023-09-01

**Authors:** Masafumi Nishino, Toshihiko Kakiuchi, Kayoko Fukuda, Masato Yoshiura

**Affiliations:** ^1^Department of Pediatrics, Faculty of Medicine, Saga University, Saga, Japan; ^2^Department of Gastroenterology, Hiramatsu Hospital, Ogi, Saga, Japan

**Keywords:** *Helicobacter pylori*, child, autoimmune gastritis, urea breath test, urease activity

## Abstract

The urea breath test (UBT) is often used to diagnose *Helicobacter pylori* infection and for its eradication. However, this text can give positive results even for other urease-active bacteria other than *H. pylori*. Even after the successful eradication *of H. pylori*, the presence of other urease-active bacteria in the gut and oral cavity can lead to positive UBT results in patients with decreased gastric acid secretion. Herein, a 15-year-old boy was diagnosed with *H. pylori* infection through the testing and treatment program for *H. pylori* for third-year junior high-school students in Saga Prefecture initiated in 2016. He underwent triple therapy comprising vonoprazan; however, UBT was found to be positive even after therapy. The results remained positive even after fourth-line eradication therapy. Stool antigen, PCR using gastric fluid, microscopy, culture, and rapid urease tests were all negative. Pepsinogen levels were normal, and none of the findings suggested autoimmune gastritis. Gastric microflora analysis revealed oral flora showing urease activity. UBT is considered useful for determining the successful eradication of *H. pylori*; however, it may give false-positive results for both *H. pylori* infection and eradication judgment. Although the patient did not have autoimmune gastritis or decreased gastric acid secretion, it is presumed that oral commensal bacteria showing urease activity inhabited the stomach, resulting in the persistently positive UBT results. In conclusion, repeated false-positive UBT results for *H. pylori* may occur even without gastric acid hyposecretion. If *H. pylori* eradication is unsuccessful based on UBT, additional test by stool *H. pylori* antigen tests should be considered.

## 1. Introduction

One of the major risk factors for gastric cancer is *Helicobacter pylori* infection (1). Prompt eradicating of *H. pylori* reduces the risk of gastric cancer (2). Accordingly, *H. pylori* screening and treatment (test and treatment) program for middle-and high-school students for the primary prevention of gastric cancer is now being take up by local governments in Japan (3).

The urea breath test (UBT) is often used to diagnose *H. pylori* (4). However, UBT is also positive for urease-active bacteria other than *H. pylori*. Even after the successful eradication of *H. pylori*, the presence of urease-active bacteria in the gut and oral cavity can lead to positive UBT results in patients with decreased gastric acid secretion, such as those with autoimmune gastritis (AIG). This is defined as “repeated false-positive UBT for *H. pylori*,” as advocated by Furuta et al. (5), which occurs when only UBT is used to determine *H pylori* eradication.

Herein, we report a pediatric case of repeated futile *H. pylori* eradication regimen because of false-positive UBT without decreased gastric acid secretion. This is the first pediatric case of this phenomenon.

## 2. Case report

**Figure 1:**
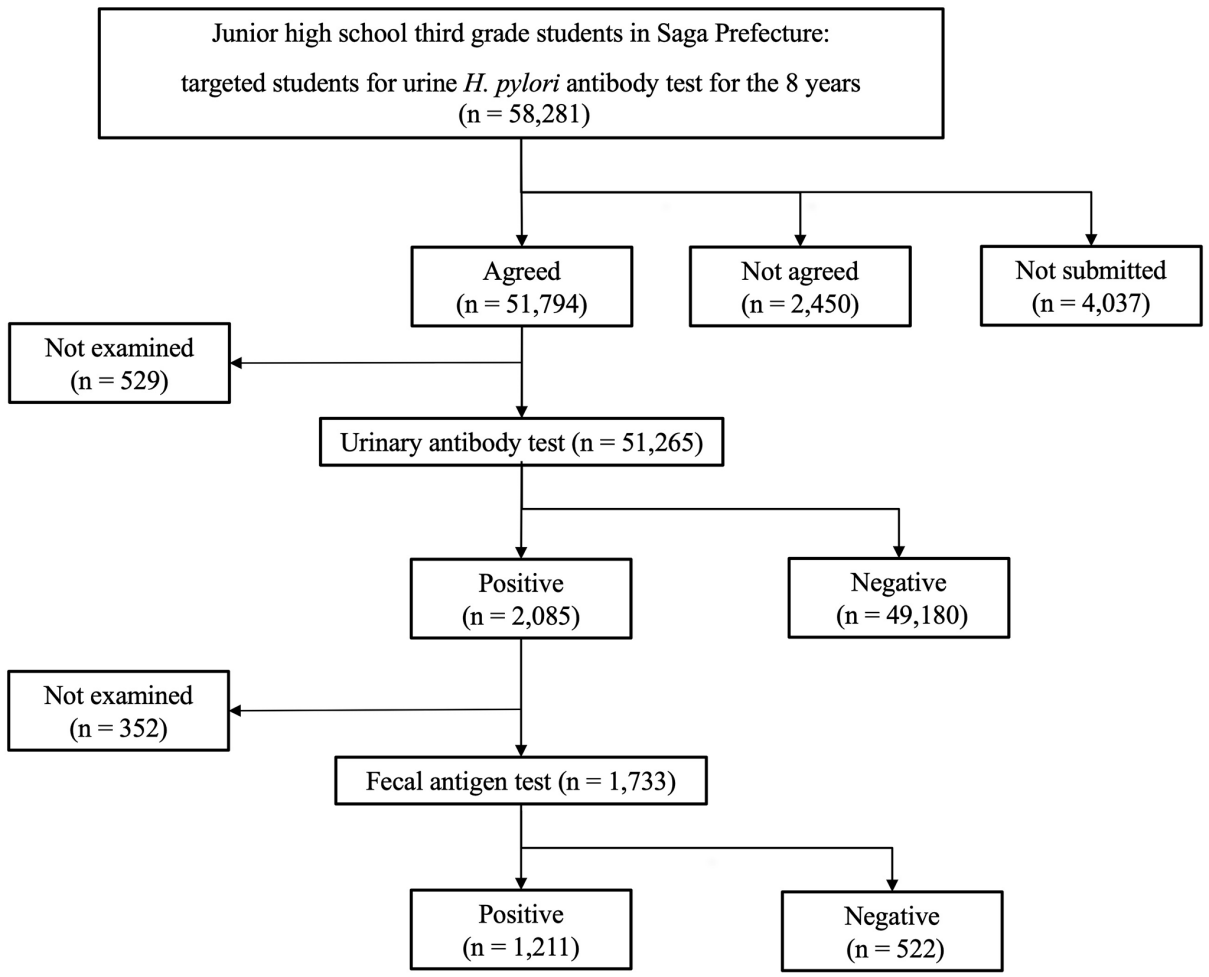
Summary for *Helicobacter pylori* screening and treatment in the Saga Prefecture for over 8 years of the study period (2016–2023). Of the total 58,281 patients, 51,265 underwent primary urinary antibody test for *H. pylori* and 1,733 underwent a secondary stool antigen test. Finally, 1,211 students had two positive tests and were diagnosed with *H. pylori* infection.

A 15-year-old boy was diagnosed with *H. pylori* infection at the test-and-treat program for *H. pylori* for third-year junior high-school students in Saga Prefecture in 2016 (6). Figure 1 presents the flowchart for the *H. pylori* test and treatment program in Saga Prefecture that is being conducted over the 8 years of the study period (2016–2023). We present the case of a patient diagnosed with *H. pylori* infection through both positive urine *H. pylori* antibody and stool antigen tests (SATs). This patient was included in the 2.0% (1,211/58, 281) *H. pylori* infection rate in our program. He exhibited no recent gastrointestinal symptoms and had no family history of *H. pylori* infection. He underwent eradication therapy for *H. pylori* at a medical institution participating in the Saga program. He was treated with a triple therapy of 20 mg vonoprazan (VPZ), 750 mg amoxicillin (AMPC), and 200 mg clarithromycin (CAM) twice daily for 7 days (Table 1). After 8 weeks, a UBT indicated that the eradication therapy was unsuccessful. He underwent second-line eradication therapy with 20 mg VPZ, 750 mg AMPC, and 250 mg metronidazole (MTZ) twice daily for 7 days. However, his UBT tests remained still positive. Third-line eradication therapy was performed with 20 mg VPZ, 750 mg AMPC, and 100 mg sitafloxacin (STFX) twice for 10 days daily. The fourth-line eradication therapy included 20 mg VPZ, 250 mg MTZ, and 100 mg STFX twice daily for 14 days. However, the UBT results after both therapies (10.2%, normal range < 2.5%) were positive, indicating eradication therapy failure. The patient was 100% compliant with all four eradication therapies and did not experience any adverse effects during or immediately after each line of therapy.

**Table 1:** *Helicobacter pylori* eradication regimens and urea breath test results.

	Regimen	Drug doses twice for day	Duration (days)	UBT results
First-line	VPZ, AMPC, CAM	20, 750, 200 mg	7	Positive (9.6‰)
Secondary-line	VPZ, AMPC, MTZ	20, 750, 250 mg	7	Positive (9.7‰)
Tertiary-line	VPZ, AMPC, STFX	20, 750, 100 mg	10	Positive (3.3‰)
Quaternary-line	VPZ, MTZ, STFX	20, 250, 100 mg	14	Positive (10.2‰)

VPZ, vonoprazan; AMPC, amoxicillin; CAM, clarithromycin; MTZ, metronidazole; STFX, sitafloxacin; UBT, urea breath test.

**Figure 2:**
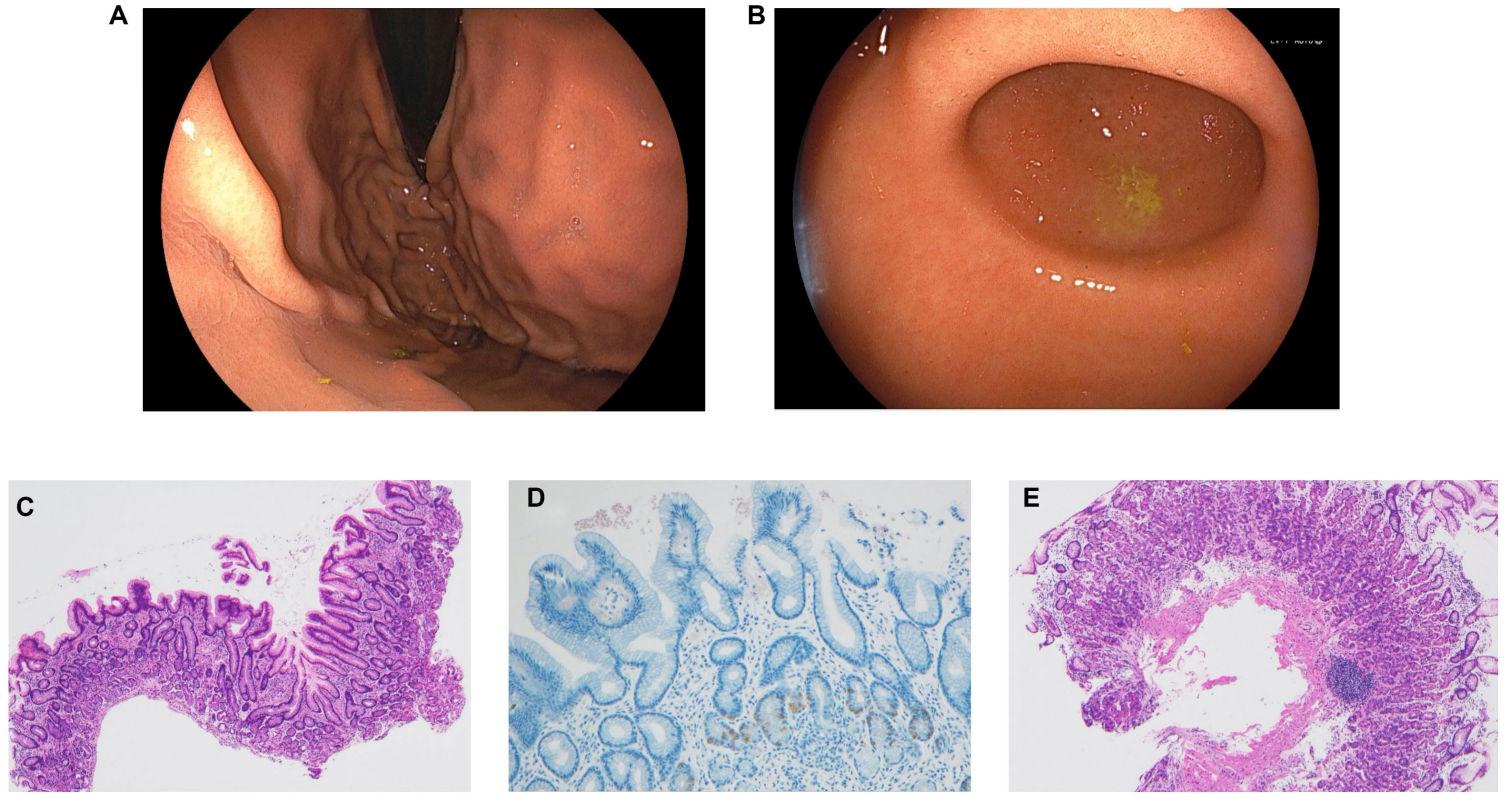
**(A)** Esophagogastroduodenoscopy revealed mild antral atrophy (Kimura–Takemoto classification C-2). **(B)** Atrophy of the fundus and corpus was not observed. None of the findings were suggestive of *H. pylori*, such as diffuse redness, mucosal swelling, patchy redness, enlarged fold, foveolar-hyperplastic polyp, nodularity, and xanthoma. **(C)** Mucosal pathological findings in the gastric antrum showed mild atrophy and intestinal metaplasia in part of the epithelium. Chronic inflammatory cell infiltration mainly comprising lymphocytes and plasma cells along with fibrosis was observed in the lamina propria (hematoxylin–eosin stain, ×40). **(D)** Gastrin immunostaining showed no evidence of positive G-cell hyperplasia (×100). **(E)** Mucosal pathological findings of the gastric corpus showed no atrophy or intestinal metaplasia of the epithelium (hematoxylin–eosin stain, ×40).

Considering the possibility of false-positive UBT results, we decided to conduct more tests. The serum *H. pylori* antibody titer was 13 U/mL (normal range, <10 U/mL), and the SATs was negative. Esophagogastroduodenoscopy (EGD) revealed mild antral atrophy (Kimura–Takemoto classification C-2; Figure 2A). Atrophy of the fundus and corpus was not observed (Figure 2B). There were no findings suggestive of *H. pylori*, such as diffuse redness, mucosal swelling, patchy redness, enlarged folds, foveolar-hyperplastic polyp, nodularity, or xanthoma. Negative results were obtained for both the rapid urease test as well as the point-of-care testing kit with intragastric fluid, a novel kit for detecting *H. pylori* and CAM resistance (7). *H. pylori* bacteria were not identified on microscopic examination with hematoxylin–eosin and Giemsa staining. A culture test for *H. pylori* using intragastric fluid was also negative. Mucosal pathology of the gastric antrum showed mild atrophy and intestinal metaplasia in a part of the epithelium. In the lamina propria, chronic inflammatory cell infiltration mainly comprising lymphocytes and plasma cells was observed, along with fibrosis (Figure 2C). Gastrin immunostaining revealed no evidence of positive G-cell hyperplasia (Figure 2D). Mucosal pathology of the gastric corpus indicated no atrophy or intestinal metaplasia of the epithelium (Figure 2E).

**Figure 3:**
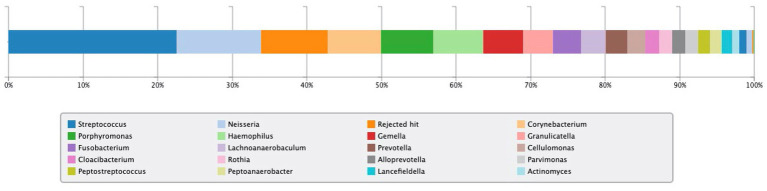
Result of 16S rRNA gastric microbiota analysis. *Neisseria* spp., *Corynebacterium* spp., *Haemophilus* spp., and *Actinomyces* spp. were detected at the genus level and have been evaluated as having urease activity.

In addition, intrinsic factor and gastric parietal cell antibody tests were negative. The level of pepsinogen 1 was within normal at 23.4 ng/mL (normal range, 15–100), and the ratio of pepsinogen 1:2 was 4.9 (normal range, >3). Gastric microbiota analysis using 16S rRNA was positive for *Neisseria* spp., *Corynebacterium* spp., *Haemophilus* spp., and *Actinomyces* spp. at the genus level (Figure 3).

## 3. Discussion

The clinical course in the present case highlights two important issues. First, repeated false-positive UBT results for *H. pylori* can also occur in children; therefore, physicians must be aware of these false-positive results while determining *H. pylori* eradication in this population. Second, paying attention to such false-positive UBT results is necessary, even in cases without gastric acid hyposecretion, such as AIG.

The current patient was diagnosed with *H. pylori* infection based on positive urine and serum antibody tests and SATs before eradication along with mild atrophy in the antrum through endoscopic and pathological findings. Other examination methods other than UBT after four eradication treatments did not indicate infection with *H. pylori*, indicating that eradication was successful at some stage during treatment. In Japan, triple therapy with VPZ is largely successful up to secondary eradication (8). This is true in both adults and children (3). In the test-and-treat program for *H. pylori* in the Saga Prefecture, the success rates of the first-and second-line eradication treatment were 83.3% (777/933) and 95.5% (128/134) over 8 years, respectively. UBT can only be used to determine the presence of urease activity in the stomach; it is not a test that directly confirms the presence of *H. pylori*. UBT is also considered useful for determining the eradication of *H. pylori* in the guidelines (9); however, as shown in this case, a positive result may be obtained even if *H. pylori* infection has been eradicated. Depending on the drug used for eradication therapy and whether a previous *H. pylori* drug resistance test was performed, if UBT reveals secondary eradication failure, it may be important to consider eradication confirmation tests other than UBT. Pediatric *H. pylori* guidelines recommend UBT or SATs to determine the success or failure of *H. pylori* eradication (9, 10). However, as mentioned in the guidelines, performing both tests is recommended. To that end, the frequency of UBT false-positive cases, like the present case, among patients with *H. pylori* eradicated must be examined because this indicates the cost effectiveness of treatment. Furuta et al. (5) described repeated false-positive UBT results for *H. pylori* in adult patients as continued positive UBT results despite the eradication of *H. pylori*. Our report indicates that such a phenomenon could also occur in children.

Although the current patient did not have AIG or exhibit decreased gastric acid secretion, oral commensal bacteria with urease activity may have inhabited the stomach, resulting in the unnecessary continuation of *H. pylori* eradication therapy. Figure 2 shows the results of gastric microflora analysis using gastric fluid at the time of UBT positivity after four rounds of eradication. In the present case, four bacteria genera (*Neisseria*, *Corynebacterium*, *Haemophilus*, and *Actinomyces*), which may have urease activity as reported by Furuta et al. (5), were detected. The problem with the present case was that it was no possible to biochemically prove whether the bacterial genera detected in the gastric flora analysis actually showed urease activity.

Gastric microbiota comprises bacteria ingested primarily through the ororespiratory tract and secondarily from the intestines by transpyloric biliary reflux (11). Persistent *H. pylori* infection decreases gastric acid secretion, which might affect the gastric microbiota in adults (12) and children (13, 14). Andersson et al. (15) revealed that *H. pylori* was the dominant bacterium whenever isolated, although its absence was associated with a diverse microbiota. This information indicates that *H. pylori* can have inhibitory effects on the colonization of other bacteria harboring a significantly lower diversity in the stomach (16). On the contrary, even without *H. pylori* infection, previous reports have suggested that the predominant phyla in the gastric mucosa include *Streptococcus*, *Rothia*, *Lactobacillus*, *Veillonella*, *Prevotella*, *Neisseria*, and *Hemophilus*, counting more than one hundred sorts (11, 17). The predominance of any of these bacteria with urease activity in the stomach could result in a false-positive UBT. Further studies investigating the prevalence of oral commensal bacteria in cases of persistent positive UBT results following *H. pylori* eradication are warranted (Figure 3).

In conclusion, repeated false-positive UBT for *H. pylori* can occur even without gastric acid hyposecretion. If UBT results are persistently positive even after *H. pylori* is eradicated multiple times, the presence of urease-activating bacteria other than *H. pylori* must be considered. Specifically, eradication must be determined by testing with both UBT and SATs.

## Data availability statement

The original contributions presented in the study are included in the article/supplementary material, further inquiries can be directed to the corresponding author.

## Ethics statement

Written informed consent was obtained from the individual(s), and minor(s)’ legal guardian/next of kin, for the publication of any potentially identifiable images or data included in this article.

## Author contributions

MN: Conceptualization, Data curation, Writing – Original draft. TK: Conceptualization, Data curation, Formal analysis, Investigation, Project administration, Supervision, Writing – Review & editing. KF: Conceptualization, Data curation, Writing – Review & editing. MY: Conceptualization, Data curation, Supervision, Writing – Review & editing.

## Funding

The author(s) declare financial support was received for the research, authorship, and/or publication of this article.

## Conflict of interest

The authors declare that the research was conducted in the absence of any commercial or financial relationships that could be construed as a potential conflict of interest.

## Publisher’s note

All claims expressed in this article are solely those of the authors and do not necessarily represent those of their affiliated organizations, or those of the publisher, the editors and the reviewers. Any product that may be evaluated in this article, or claim that may be made by its manufacturer, is not guaranteed or endorsed by the publisher.
